# SAMHD1 Restricts HIV-1 Replication and Regulates Interferon Production in Mouse Myeloid Cells

**DOI:** 10.1371/journal.pone.0089558

**Published:** 2014-02-19

**Authors:** Ruonan Zhang, Nicolin Bloch, Laura A. Nguyen, Baek Kim, Nathaniel R. Landau

**Affiliations:** 1 Department of Microbiology, NYU School of Medicine, New York, New York, United States of America; 2 Department of Pathology, University of Rochester, Rochester, New York, United States of America; 3 Center for Drug Discovery, Department of Pediatrics, Emory School of Medicine, Atlanta, Georgia, United States of America; Temple University School of Medicine, United States of America

## Abstract

SAMHD1 restricts the replication of HIV-1 and other retroviruses in human myeloid and resting CD4^+^ T cells and that is counteracted in SIV and HIV-2 by the Vpx accessory protein. The protein is a phosphohydrolase that lowers the concentration of deoxynucleoside triphosphates (dNTP), blocking reverse transcription of the viral RNA genome. Polymorphisms in the gene encoding SAMHD1 are associated with Aicardi-Goutières Syndrome, a neurological disorder characterized by increased type-I interferon production. SAMHD1 is conserved in mammals but its role in restricting virus replication and controlling interferon production in non-primate species is not well understood. We show that SAMHD1 is catalytically active and expressed at high levels in mouse spleen, lymph nodes, thymus and lung. siRNA knock-down of SAMHD1 in bone marrow-derived macrophages increased their susceptibility to HIV-1 infection. shRNA knock-down of SAMHD1 in the murine monocytic cell-line RAW264.7 increased its susceptibility to HIV-1 and murine leukemia virus and increased the levels of the dNTP pool. In addition, SAMHD1 knock-down in RAW264.7 cells induced the production of type-I interferon and several interferon-stimulated genes, modeling the situation in Aicardi-Goutières Syndrome. Our findings suggest that the role of SAMHD1 in restricting viruses is conserved in the mouse. The RAW264.7 cell-line serves as a useful tool to study the antiviral and innate immune response functions of SAMHD1.

## Introduction

Human cells express several proteins that restrict the replication of viruses such as human immunodeficiency virus type 1 (HIV-1). One such protein is SAM and HD domain 1 protein (SAMHD1), a phosphohydrolase that is expressed in monocyte derived-dendritic cells (MDDC), monocyte-derived macrophages (MDM) and resting T cells where it blocks the infection of retroviruses at early reverse transcription. SAMHD1 is a dGTP-regulated triphosphohydrolase that removes the triphosphate from deoxynucleoside triphosphates (dNTP), depleting the pool of the deoxynucleotide precursors that are needed to synthesize the virus DNA from viral RNA genome [1]–[6]. In addition to inhibiting HIV-1, SAMHD1 blocks the replication of a broad range of retroviruses including murine leukemia virus (MLV) and DNA viruses such as herpes simplex virus type 1 and vaccinia virus [1], [2], [7]–[10]. In MDM and resting T cells, the block can be partially relieved by the addition to the culture medium of deoxynucleosides (dN) that are converted through the salvage pathway to dNTP, restoring the intracellular dNTP pool [5], [7], [11].

In HIV-2, simian immunodeficiency virus (SIV) of sooty mangabeys, SIV of macaques (SIVmac) and related lentiviruses, SAMHD1 is counteracted by the viral accessory protein Vpx [1], [2], [12]. In SIVs such as the SIV of African green monkeys, the ability to counteract SAMHD1 is accomplished by Vpr, a related virion-packaged accessory protein [13]. Vpx and Vpr are virion-packaged proteins that are released into the cytoplasm of the target cell post-entry whereupon they bind SAMHD1 to induce its degradation by recruiting the cullin4A-RING E3 ubiquitin ligase complex CRL4. SAMHD1 is localized to the nucleus of the cell through a nuclear localization sequence located at amino acids 11–14 and its degradation is thought to occur in the nucleus through the activity of nuclear CRL4 [14]–[17]. HIV-1 does not encode Vpx and its Vpr does not target SAMHD1 for degradation. As a result, HIV-1 replication in myeloid cells is attenuated.

The mechanisms that regulate the antiviral activity of SAMHD1 in cells are not well understood. Although SAMHD1 is expressed in myeloid cells and T cells, it lacks antiviral activity in actively replicating CD4^+^ T cells, transformed lymphoid cell-lines and cycling monocytic cell-lines. The antiviral activity of SAMHD1 is regulated by phosphorylation of T592 by CDK1 in cycling cells. T592 is dephosphorylated in nondividing, terminally differentiated cells where it has antiviral activity [18], [19]. Mutation of T592 to the phosphomimetic aspartic or glutamic acid inactivates the antiviral activity of SAMHD1 while mutation to alanine or valine has no effect, suggesting that the antiviral activity of SAMHD1 is shut off in cycling cells by phosphorylation at T592. Paradoxically, T592D and T592E mutants retain phosphohydrolase activity [18], a finding that suggests that dNTP pool depletion does not fully account for the mechanism by which SAMHD1 restricts virus replication. *E. coli*-produced recombinant SAMHD1 is reported to have exonuclease activity, raising the possibility of restriction by nucleolytic attack of the viral reverse transcript [20].

Vpx is packaged into SIV virions during virus assembly by an interaction with an amino acid motif in p6 Gag. Because HIV-1 lacks the Vpx packaging motif it does not package Vpx [21], [22]. Placement of the SIVmac Vpx-packaging motif into the p6 region of HIV-1 Gag allows the virus to package Vpx provided *in trans* [22], [23]. The infection of MDDC, MDM and resting T cells by HIV-1 can be promoted either by infecting the cells with Vpx-containing virions or by pre-treating the cells with Vpx-containing virus-like particles [24]. MDDCs infected with HIV-1 as the result of Vpx activate innate immune response pathways as characterized by the production of type-I interferon (IFN) and are induced to mature [25]. The cells sense the infection through a yet unidentified cytoplasmic sensor that responds to newly produced Gag protein [25].

In humans, polymorphisms in the SAMHD1 gene that introduce premature terminations in the open reading frame or missense mutations that inactivate phosphohydrolase activity are associated with Aicardi-Goutières syndrome (AGS), an early-onset neurological condition characterized by chronically elevated levels of type-I IFN in the cerebrospinal fluid [26]. AGS is also associated with polymorphisms in the genes encoding TREX1 [27], RNase H2 subunits [28] and ADAR1 [29]. TREX1 and ADAR1 suppress the induction of type-I IFN by preventing viral nucleic acid accumulation [30], [31]. The mechanism by which defects in SAMHD1 elevate type-I IFN levels is not understood.

SAMHD1 was first identified in the mouse as a type-II IFN inducible gene [32]. The human homologue was found to be type-I IFN-inducible in a tissue-specific manner [33], [34]. Type I IFN induces SAMHD1 in monocytes, HEK293T and HeLa cells but does not induce SAMHD1 in MDM [11], [35], MDDC [34] or myeloid and plasmacytoid dendritic cells (DC) [36]. In addition, type-II IFN does not induce SAMHD1 in MDM or MDDC [34], [35].

The mouse genome encodes a SAMHD1 homologue but whether the protein acts *in vivo* to restrict retroviruses is not known. Mice are not infected by lentiviruses but are subject to infection by α, β and γ retroviruses and over the course of evolution, have been host to retroviruses that have left remnants as endogenous viruses in the genome. While mouse SAMHD1 restricts retroviruses when expressed in human cells, the role of the protein in the mouse is not known.

Recently, two groups reported findings on SAMHD1 knock-out mice. In one report, HIV-1 replication was enhanced in the knock-out mice, but in the other, only an attenuated form of the virus was affected [37], [38]. To further understand the role of SAMHD1 in the mouse, we tested the effect of SAMHD1 knock-down in primary mouse macrophages on HIV-1 and murine leukemia virus (MLV) infection. Using a specific mouse anti-SAMHD1 antiserum, we find that SAMHD1 is specifically expressed in mouse myeloid and lymphoid cells and is catalytically active. Knock-down of SAMHD1 by siRNA and shRNA in primary bone marrow-derived (BMDM) and the monocytic cell-line RAW264.7 increased their infectabilty by HIV-1 and MLV. SAMHD1 knock-down in RAW264.7 induced the production of type-I IFN and IFN-stimulated genes (ISGs), mimicking human AGS.

## Materials and Methods

### Ethics Statement

Anti-SAMHD1 antibodies were prepared by Pocono Rabbit Farm and Laboratories (PRF&L), under protocol PRF2A approved by PRF&L Institutional Animal Care and Use Committee (IACUC). PRF&L is fully accredited by the Association for Assessment and Accreditation of Laboratory Animal Care and by the NIH Office of Laboratory Animal Welfare, assurance number A3886-01 expiration January 31, 2017. The experiment using mouse bone marrow cells was carried out in accordance with the recommendations in the guide for the Care and Use of Laboratory Animals of the NIH. The protocol was approved by the NYU IACUC (Laboratory Animal Protocol Number 110120-03).

### Cell culture

Bone marrow cells were flushed from the femurs of C57BL/6 mice. Contaminating red blood cells were removed by washing the cells in ammonium chloride/potassium chloride lysing buffer (Lonza). The cells were differentiated into bone marrow-derived macrophages (BMDM) by culturing for 7 days in Dulbecco’s modified Eagle medium (DMEM) supplemented with 10% fetal bovine serum (FBS), 2 mM L-glutamine and murine macrophage colony stimulating factor (M-CSF). The BMDM were maintained in M-CSF-containing medium for up to 14 days. RAW264.7, HEK293T, L929-ISRE reporter cells were cultured in DMEM supplemented with 10% FBS. Mouse splenocytes from C57BL/6 mice were stained with phycoerythrin-conjugated anti-CD3 and fluorescein isothiocyanate-conjugated anti-CD19 (BioLegend) and sorted on a BD Biosciences FACS-Aria to obtain CD3^+^ T cells and CD19^+^ B cells. T cells and B cells from the spleen were activated with 5 µg/ml concanavalin A (Con A) or 25 µg/ml lipopolysaccharide (LPS), respectively.

### Stable SAMHD1 knock-down cell-lines

Lentiviral vectors encoding shRNA that target 4 regions of the mouse SAMHD1 mRNA transcript were constructed by cloning oligonucleotide consisting of cohesive ends to Age-I (5'-end) and EcoR-I (3'-end), a 21mer hairpin sense sequence (shRNA1: 5'-GCCGCAATCTATACAAGTATT-3' ; shRNA2: 5'-CCCTCTCCTTATCAGAATCAT-3' ; shRNA3: 5'-CGACGTAGACAAATGGGATTA-3' ; shRNA4: 5'-GCAGACCCCTACGTGGAGATT-3'), a CTCGAG loop sequence, and a hairpin anti-sense sequence into the pLKO.1 *puro* vector at its Age-I and EcoR-I restriction sites. Non-targeting pLKO.1 shRNA (Sigma, SHC002) was used as a control [17]. Lentivirus stocks were prepared as vesicular stomatitis virus G (VSV-G) pseudotypes by transfecting HEK293T cells with pLKO.1 vector, pCMV ΔR89 and pcVSV-G at a ratio of 4:3:1 using Lipofectamine 2000 (Invitrogen). RAW264.7 cells (1.0×10^6^) were transduced in a 6-well dish with 1.0 ml of the virus and selected in medium containing 8.0 µg/ml puromycin 3 days later.

### siRNA knock-down in mouse BMDM

BMDM were detached from the dish by incubation in PBS/5 mM EDTA and seeded at 0.5×10^6^ cells/well in a 6-well plate. The cells were immediately transfected with 50 nM siRNA pool or individual siRNA targeting mouse SAMHD1 (Dharmacon) using HiPerfect lipofection reagent (Qiagen). The efficiency of knock-down was assessed 4 days post-transfection by immunoblot analysis.

### Virus preparation and infections

HIV-1 reporter viruses were produced by calcium phosphate cotransfection of HEK293T cells with a reporter virus plasmid (pNL-Luc or pHIV-1-CMV-GFP) and with pcVSV-G [39]–[41]. MLV reporter virus was generated by cotransfection of HEK293T cells with pMX-sfi-GFP, pHIT60 and pcVSV-G at a ratio of 4:2:1 [42], [43]. The medium was changed 6 hours post-transfection. 48 hours post-transfection, the supernatant was harvested and filtered through a 0.45 µm filter. HIV reporter viruses were concentrated 10-fold by ultracentrifugation through a 20% sucrose cushion at 30,000 rpm for 90 minutes at 4°C and frozen in aliquots. HIV-1-CMV-GFP and MLV-GFP titers were determined by infection of HEK293T cells and defined as the number of GFP-forming units (GFU) per ml. NL-Luc titer was determined by infection of HEK293T cells and defined as the number of counts per second (cps) per ml. HIV-1-CMV-GFP and MLV-GFP titers were 2.6×10^8^ GFU/ml and 8.5×10^6^ GFU/ml, respectively, and the NL-Luc titer was 3.6×10^8^ cps/ml. RAW264.7 and BMDM were seeded at 0.5×10^6^ cells/well in a 6-well plate and infected at the indicated multiplicity of infection (MOI). 72 hours post-infection, GFP reporter virus-infected cells were analyzed on a BD Biosciences LSRII flow cytometer and the data were analyzed with FlowJo software (Treestar). Luciferase reporter virus-infected cells were lysed and the luciferase activity was measured using the SteadyLite Plus reagent (PerkinElmer).

### Production of rabbit anti-mouse SAMHD1 antiserum

Full-length mouse SAMHD1 cDNA isoform 2 (ISF 2) was amplified by PCR with primers containing EcoR-I and Xho-I cleavage sites and was cloned into pET-42a(+) in-frame with a glutathione S-transferase (GST) tag. BL21 *E. coli* were transformed with the plasmid and a single colony was cultured overnight then used to seed 500 ml of Luria broth. The culture was grown for 4 hours at 37°C then induced for 3 hours with 100 µM IPTG at 30°C. The bacteria were sonicated and lysed in PBS containing 1% Triton X-100. The lysate was treated with benzonuclease then clarified by centrifugation. The SAMHD1-GST fusion protein was purified on glutathione beads (GE Healthcare) and cleaved on the beads with Factor Xa. The protein that was released was concentrated on a 10 kDa Amicon centrifugal filter and used to immunize 2 rabbits (PRF&L).

### Immunoblot analysis

Cells or homogenized mouse tissues were lysed in radio-immunoprecipitation assay buffer (50 mM Tris, pH 8, 150 mM NaCl, 1% Triton X-100, 0.5% sodium deoxycholate, 0.1% SDS) containing Halt Protease Inhibitor (ThermoScientific). Protein lysates (5 µg) were separated by 4–12% Bis-Tris SDS-PAGE gel and transferred to a polyvinylidene difluoride membrane (Invitrogen). Membranes were probed with 1∶1000 rabbit anti-mouse SAMHD1 antiserum, 1∶40,000 anti-GAPDH antibody (Ambion) and 1∶1000 anti-human SAMHD1 antibody (Origene) followed by 1∶4,000 horseradish peroxidase (HRP)-conjugated goat anti-rabbit immunoglobulin serum and 1∶40,000 HRP-conjugated goat anti-mouse immunoglobulin serum (Sigma). Membranes were developed with chemiluminescent substrate (Pierce) exposed to Hyperfilm (GE Healthcare). Band intensities were quantified using ImageJ software [44].

### dNTP pool quantification

dATP was quantified as previously described [45]. Briefly, 2.0×10^6^ cells were washed with cold PBS, lysed in 200 µl ice cold 65% methanol, heated to 95°C for 3 minutes, clarified by centrifugation for 3 minutes at 14,000 rpm, dried and resuspended in 20 µl ddH_2_O. The lysate was incubated in a reaction containing 200 fM double-stranded oligonucleotide duplex consisting of the 18 mer deoxynucleotide ^32^P-5′-GTCCCTGTTCGGGCGCCA-3′ and the complementary deoxynucleotide 19-mer 5′-TTGGCGCCCGAACAGGGAC-3′, 4 µl HIV-1 reverse transcriptase, 25 mM Tris-HCl pH 8.0, 2 mM dithiothreitol, 100 mM KCl, 5 mM MgCl_2_, and 10 µM oligo(dT). The reaction was incubated 5 minutes at 37°C, quenched 5 minutes with 10 µl 40 mM EDTA and 99% formamide at 95°C. The products were resolved on a 14% urea-PAGE gel and analyzed on a phosphoimager using QuantityOne software. Sample volumes were adjusted to ensure that the quantified dNTP content was within the linear range of the assay.

### *In vitro* phosphohydrolase assay

Cells were lysed in lysis buffer containing 50 mM HEPES, 150 mM KCl, 2 mM EDTA and 0.5% NP40 and the protein concentration was determined by Bradford assay. 1.0 mg or 0.1 mg lysate was precleared for 30 minutes at 4°C with 30 µl protein A-sepharose beads (GE healthcare). The beads (25 µl) were coated with 10 µl rabbit-anti-GST antiserum (control) or rabbit anti-mouse SAMHD1 antiserum for 30 minutes at 4°C and then washed 3 times with lysis buffer. The coated beads were added to the precleared cell lysate and incubated for 2 hours at 4°C. A quarter of the beads were resuspended in reducing sample loading buffer and the eluted protein was analyzed by immunoblot using an anti-SAMHD1 antibody (Origene). The remaining beads were incubated for 4 hours at 37°C in assay buffer (250 mM Tris pH 8.0, 250 mM KCl, 25 mM MgCl_2_, 0.5% Triton X-100) containing 0.2 µCi of [α-^32^P] dATP, 0.4 mM unlabeled dATP and 0.4 mM unlabeled dGTP. The enzyme was inactivated for 5 minutes at 70°C, the reaction products were separated on a cellulose 300 polyethylenimine thin layer chromatography (TLC) plate (Sorbent Technologies) in TLC running buffer (1 M LiCl, 0.5 M formic acid) and visualized on a Typhoon Trio Phosphorimager (GE Healthcare).

### Quantitative real-time PCR (qRT-PCR)

RAW264.7 cells expressing control or anti-SAMHD1 shRNA were seeded at 0.5×10^6^/well in a 12-well plate. 48 hours later, the cells were harvested and total RNA was prepared using TRIzol (Invitrogen). cDNA was synthesized and analyzed by qRT-PCR in triplicate with a Prism 7300 (Applied Biosystems) using SYBR green mix (Roche) and primers to mouse IFNβ, TNFα, IP-10, MX-1, IFIT-1, Trim 5, GAPDH, SAMHD1 (both isoforms), SAMHD1 ISF1 and SAMHD1 ISF2. The primer pairs used for qRT-PCR were: IFN β (5′-CCTGGAGCAGCTGAATGGAA-3′ and 5′-CCACCCAGTGCTGGAGAAAT-3′), TNFα (5′-AGGCACTCCCCCAAAAGATG-3′ and 5′-TGAGGGTCTGGGCCATAGAA-3′), IP-10 ( 5′-ATGACGGGCCAGTGAGAATG-3′ and 5′-TCAACACGTGGG CAGGAT AG-3′), MX-1 (5′-ACCCTGAAGGGGATAGGACC-3′and 5′-GCTGACCTCTGCACTTGACT-3′), IFIT-1 (5′-GGCAGAAGCCCAGATCTACC-3′ and 5′-GGCTCCACTTTCAGAGCCTT-3′), Trim 5 (5′-CATCTGCTGGCTTTGTGAGC-3′and 5′-CTGCAGCTGCTCCTTGTACT-3′), GAPDH (5′-GGATCTGACGTGCCGCCTGG-3′ and 5′-CAGCCCCGGCATCGA AGGTG-3′), SAMHD1 (both isoforms) (5′-CTGGTGCGAGCACTTGCCGA-3′ and 5′-TGGGCGAGCCCGTGGGATAA-3′), SAMHD1 ISF1 (5′-CAAGCCACAGGATGGTGA CA-3′ and 5′-TCTTGGAGGCAAGATGAAGTC T-3′), SAMHD1 ISF2 (5′-CAAGCCACAGCAATGTGGGGCT-3′ and 5′-CTGGTTGTGAGCCGCATGT-3′). C_t_ values were calculated and normalized to GAPDH.

### IFNβ measurement

L929-ISRE luciferase reporter cells (1×10^5^ cells/well) were seeded in a 96-well plate. The next day, 200 µl of cell supernatant was added to each well. 6 hours later, the cells were lysed and luciferase activity was measured using SteadyLite Plus reagent. The concentration of IFNβ was calculated based on a standard curve generated using recombinant mouse IFNβ.

## Results

### SAMHD1 is expressed in lymphoid and myeloid cells in the mouse

**Figure 1:**
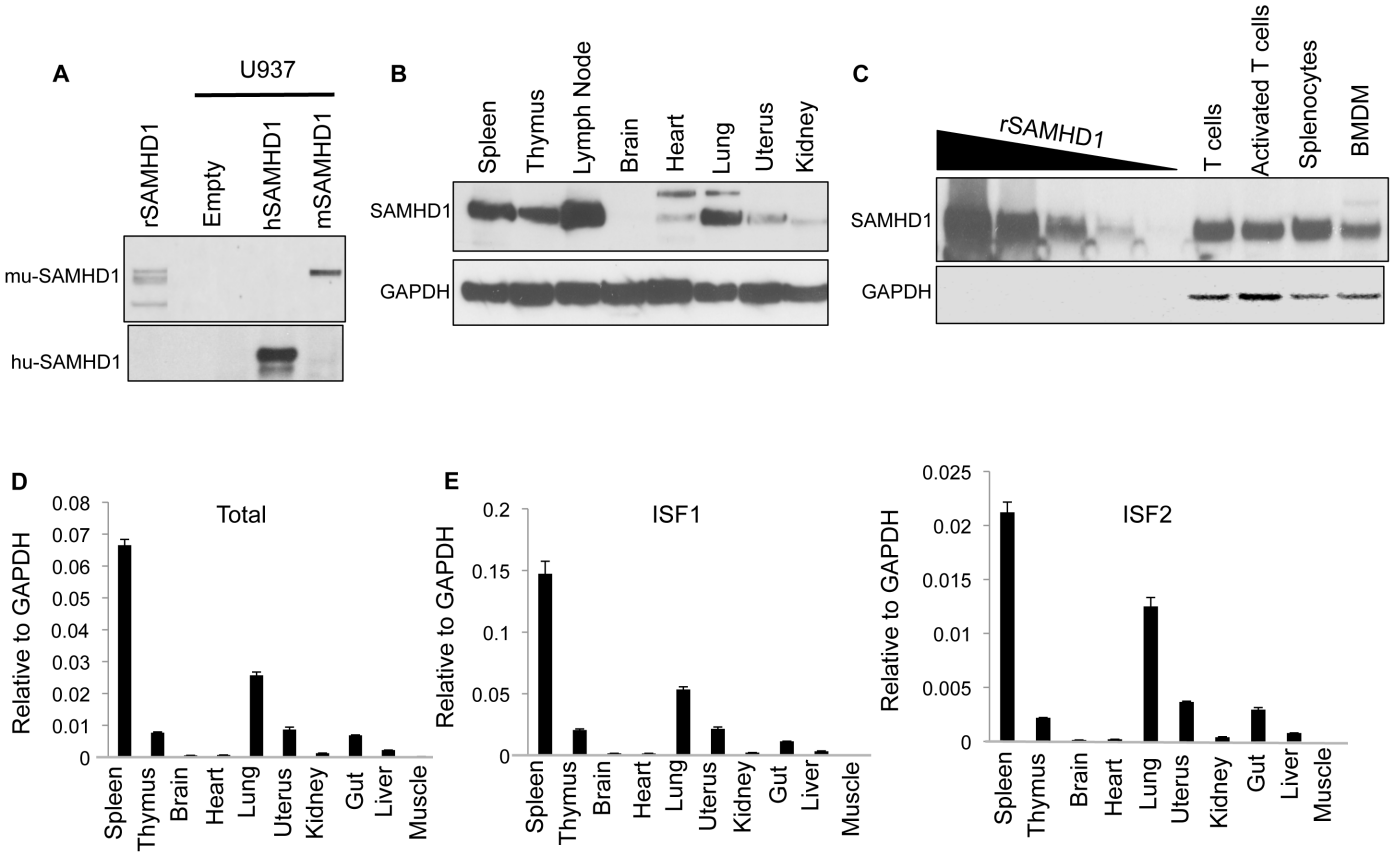
SAMHD1 expression in various mouse tissues. (A) Recombinant mouse SAMHD1 (rSAMHD1) and lysates from parental U937 or U937 expressing human or mouse SAMHD1 were analyzed on an immunoblot probed with rabbit anti-mouse SAMHD1 ISF2 antiserum or with anti-human SAMHD1 antibody. (B) SAMHD1 in lysates of mouse tissues was analyzed on an immunoblot probed with rabbit anti-mouse SAMHD1 antiserum and anti-GAPDH as a loading control. (C) 1000, 200, 40, 8 and 1.6 ng of recombinant mouse SAMHD1 protein and 15 µg of protein from cell lysates from mouse T cells, Con-A-activated T cells, splenocytes and BMDM were analyzed on an immunoblot probed with rabbit anti-SAMHD1 antiserum and anti-GAPDH as a loading control. (D) RNA was extracted from homogenized mouse tissues, and SAMHD1 expression was determined in triplicate as the ratio of SAMHD1 to GAPDH by qRT-PCR using primers recognizing both SAMHD1 isoforms. (E) ISF1 and ISF2 transcript levels were determined in triplicate as their ratio to GAPDH by qRT-PCR using isoform-specific primers. The data are representative of two experiments in A-C. Measurements were in triplicate in D and E and the error bars indicate the standard deviation of the mean.

In humans, SAMHD1 is expressed in monocytes, macrophages, DCs, resting and activated T lymphocytes and astrocytes. To determine the tissue expression in the mouse, we generated a rabbit antiserum against purified *E. coli*-produced recombinant mouse SAMHD1. The antiserum sensitively detected recombinant SAMHD1 and specifically detected mouse SAMHD1 expressed in transduced U937 cells but not human SAMHD1 (Fig. 1A). To determine the tissue distribution of mouse SAMHD1 we analyzed lysates of primary mouse tissues on an immunoblot probed with the rabbit antiserum. SAMHD1 was highly expressed in mouse spleen, thymus, lymph nodes and lung (Fig. 1B). It was not detectably expressed, or expressed at low level in the brain, heart, uterus and kidney. In lung, expression was most likely from alveolar macrophages. The absence of the protein from the brain was surprising as this tissue contains macrophage-like microglial cells that would be expected to express SAMHD1.

To further characterize SAMHD1 expression, we analyzed resting and activated T cells, splenocytes and BMDM. SAMHD1 was expressed at a similar level in resting and activated T cells, splenocytes and BMDM. By comparing band intensities in the tissue lysates to those of a standard curve using the recombinant mouse protein we were able to determine the number of SAMHD1 molecules per cell (Fig. 1C). According to this calculation, 15 µg T cell lysate on a single lane of the immunoblot was derived from 0.9×10^6^ cells. The band for SAMHD1 contained 100 ng of protein based on the recombinant SAMHD1 standard curve. At a molecular mass of 144 kDa for SAMHD1 dimers, this yields 4.7×10^5^ molecules of SAMHD1 per T cell.

Human SAMHD1 is expressed from a single spliced mRNA. In the mouse, two transcripts are generated that differ by alternative splicing of the last exon, termed isoform 1 (ISF1) and isoform 2 (ISF2). ISF1 and ISF2 are identical up to amino acid 624 and then diverge at the carboxy-terminal 34 and 27 amino acids, respectively. Both isoforms diminish the dNTP pool and restrict HIV-1 infection when expressed in the human monocytic cell-line U937 [5]. To determine the relative tissue distribution of the two isoforms, we collected RNA from mouse tissues and quantified the mRNA transcripts by qRT-PCR using primers that detected both isoforms or were isoform-specific (Fig. 1E). The results showed that SAMHD1 expression was the highest in spleen, moderate in thymus, lung, uterus and gut, and undetectable in brain, heart, kidney, liver and muscle (Fig. 1D and E). ISF1 was present at a 7-fold higher copy number than ISF2 in all of the tissues analyzed.

### SAMHD1 is catalytically active in resting and activated T and B cells, BMDM, RAW264.7 cells and splenocytes

**Figure 2:**
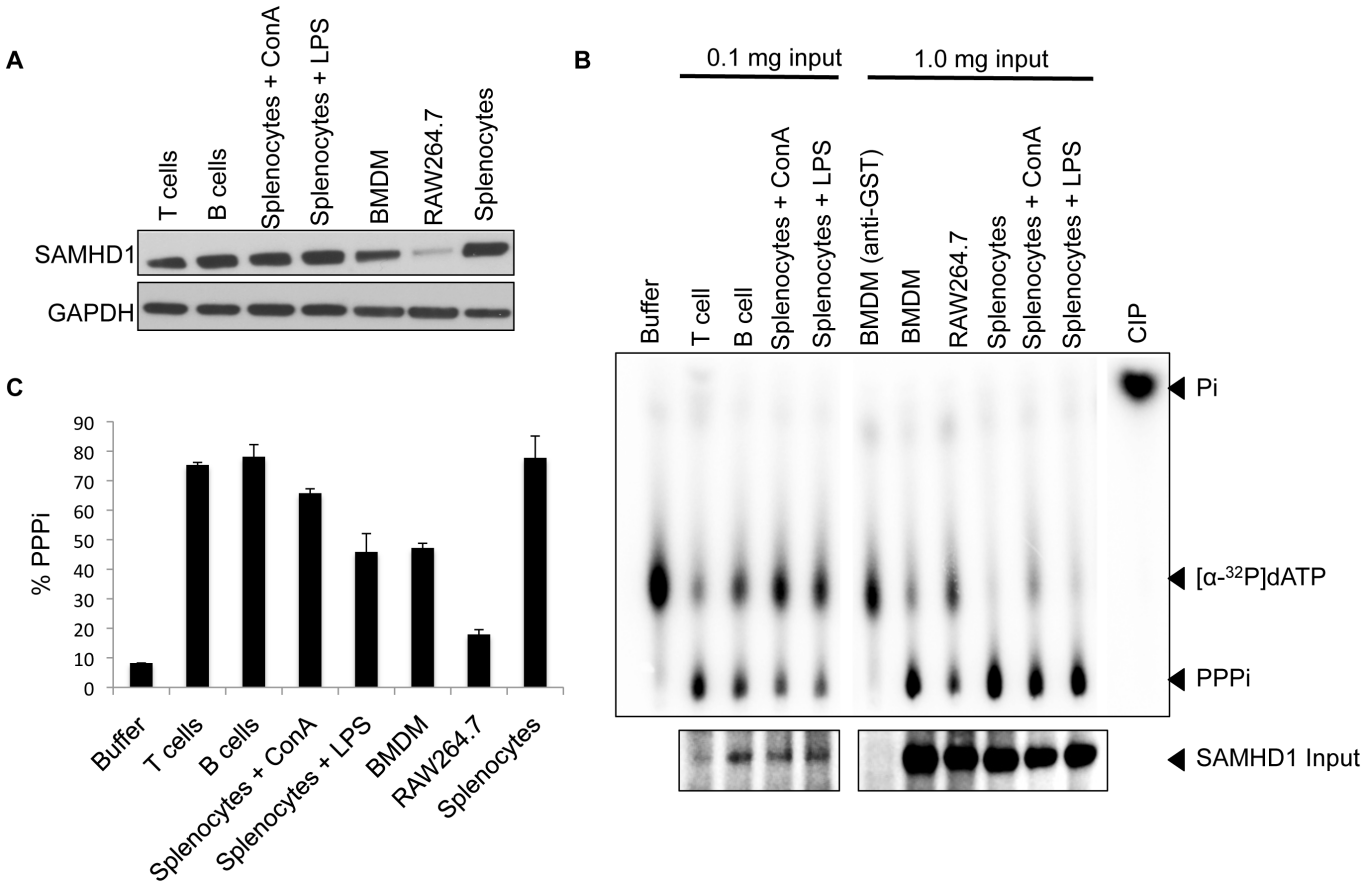
SAMHD1 in primary mouse cells is catalytically active. (A) Mouse T cells, B cells, Con A-activated T cells and LPS-activated B cells from splenocytes, BMDM, RAW264.7 cells and splenocytes were lysed, and SAMHD1 and GAPDH loading control were detected on an immunoblot. (B) SAMHD1 was immunoprecipitated from 0.1 mg or 1.0 mg cell lysates and incubated with [α-^32^P]-dATP for 240 minutes. The products were visualized by TLC and autoradiography (top panel). Controls included a reaction with buffer alone (buffer), BMDM lysate immunoprecipitated with anti-GST antiserum and a calf intestinal phosphate (CIP)-treated reaction. 10% of the input protein was analyzed on an immunoblot (bottom panel). (C) The catalytic activity of SAMHD1 immunoprecipitated from 70 µg cell lysates was quantified. Measurements were made in duplicate and the error bars indicate the standard deviation of the mean.

The regulation of SAMHD1 catalytic activity has not been explored. To test whether the phosphohydrolase activity of SAMHD1 might differ in various mouse cells and tissues, we pulled-down SAMHD1 from resting and activated mouse T and B cells, BMDM, total splenocytes and from the Abelson murine leukemia virus-transformed mouse macrophage cell line RAW264.7 and then tested their catalytic activity using an *in vitro* phosphohydrolase assay. Immunoblot analysis confirmed that SAMHD1 was expressed in each primary cell-type. RAW264.7 cells also expressed SAMHD1, although at a lower level than the primary cells (Fig. 2A). For the phosphohydrolase assay, we immunoprecipitated SAMHD1 from 0.1 mg or 1.0 mg cell lysates, incubated the immunoprecipitates with [α-^32^P] labeled dATP and detected the products by autoradiography after separation on TLC. The results showed that SAMHD1 was catalytically active in all of the cell-types and its activity was not affected by activation state (Fig. 2B). We then quantified the catalytic activity of SAMHD1 immunoprecipitated from 70 µg cell lysate. The catalytic activity of SAMHD1 in RAW264.7 was less than that of the primary cells in accord with its lower abundance (Fig. 2C).

### Knock-down of SAMHD1 in RAW 264.7 increases the level of the dNTP pool and enhances retrovirus infection

**Figure 3:**
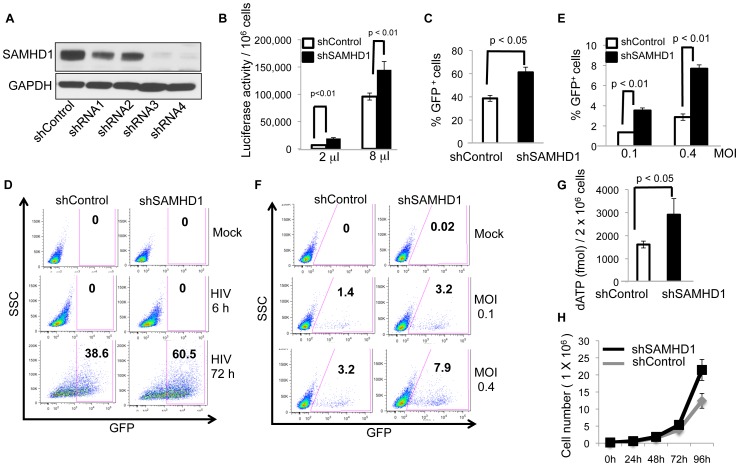
shRNA knock-down of SAMHD1 in the RAW264.7 cell-line enhances retroviral infection. (A) Cell lysates from RAW264.7 expressing control shRNA or anti-SAMHD1 shRNA were analyzed by immunoblot using rabbit anti-SAMHD1 antiserum with GAPDH detected as a loading control. (B) SAMHD1 knock-down RAW264.7 cells and control cells were infected with 2 µl (7.2×10^5^ cps) and 8 µl (28.8×10^5^ cps) NL-Luc reporter virus and after 3 days, luciferase activity was measured. (C, D) RAW264.7 cells were infected at MOI = 4 with HIV-1-CMV-GFP reporter virus and analyzed 3 days later by flow cytometry. The average of 3 independent experiments is shown in (C). One representative flow cytometry plot for each condition is shown in (D). Cells infected for 6 hours served as a control for baseline fluorescence. (E, F) RAW264.7 cells were infected with MLV-GFP at MOI = 0.1 and 0.4. Three days later, the cells were analyzed by flow cytometry. The average of 3 independent experiments is shown in (E). One representative flow cytometry plot for each condition is shown in (F). (G) The dNTP pool of SAMHD1 knock-down and control RAW264.7 cells was quantified by single-nucleotide extension assay. (H) The growth of SAMHD1 knock-down and control RAW264.7 cells was measured by counting cell numbers over 4 days. SAMHD1 shRNA3 was used in (B-F). Measurements were in triplicate with error bars to indicate the standard deviation of the mean. Statistical significance was calculated using the student’s t-test.

Mouse SAMHD1 has antiviral activity when expressed in U937 cells but its role *in vivo* is not known. To test whether mouse SAMHD1 restricts lentiviral and retroviral infection, we stably knocked down SAMHD1 in RAW264.7 cells by transducing separately with 4 different shRNA lentiviral vectors, each targeting a different sequence in the SAMHD1 mRNA transcript and, as a control, established a cell-line transduced with a non-targeting shRNA (shControl). To determine the knock-down efficiency, we quantified SAMHD1 by immunoblot. All 4 shRNAs decreased the amount of SAMHD1 protein in the cells with shRNA3 and shRNA4 being the most effective (Fig. 3A). To determine the effect of SAMHD1 knock-down on infectability of the cells, we challenged them with luciferase and GFP HIV-1 reporter viruses. SAMHD1 knock-down caused a 1.5-2.8-fold increase in luciferase reporter virus infection (Fig. 3B) and a 1.6-fold increase in the number of cells infected by the GFP reporter virus (Fig. 3C, D). To determine the effect of SAMHD1 knock-down on murine retrovirus, we infected the cells with MLV-GFP. We found that SAMHD1 knock-down increased the number of infected cells 2.6-fold (Fig. 3E, F). While the increases in infectability of HIV-1 and MLV were modest, they were reproducible in three repetitions of the experiment. The results suggested that SAMHD1 can have a least a small effect on virus replication in dividing cells.

To determine whether the effect might be caused by changes in the size of the dNTP pool, we quantified dATP by a single nucleotide extension assay in the RAW264.7 cells [45]. We found that SAMHD1 knock-down increased the dNTP pool 1.8-fold (Fig. 3G). While this difference was small, it was reproducible in three repetitions of the experiments and was similar to the effect on virus replication. During the course of the experiments, we noticed that the SAMHD1 knock-down cells grew somewhat faster than the control cells. To quantify this difference, we measured the growth rates of the SAMHD1 knock-down and control cells over 4 days. We found that the SAMHD1 knock-down cells grew slightly faster than the control cells suggesting that regulation of the dNTP pool by SAMHD1 can affect the growth-rate of cells (Fig. 3H).

### SAMHD1 restricts retroviral infection and depletes the dNTP pool in mouse BMDM

**Figure 4:**
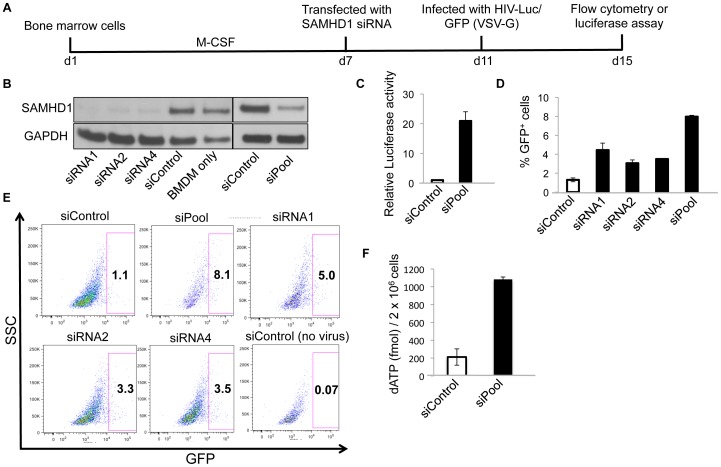
Knock-down of SAMHD1 in BMDM increases HIV-1 infection. (A) The time-line shows the scheme for determining the effect of SAMHD1 knock-down on HIV-1 infection of primary BMDM. Bone marrow cells were differentiated to BMDM in M-CSF and then transfected with siRNA. The cells were then infected with luciferase or GFP reporter virus and the infection was quantified after 4 days. (B) BMDM were transfected with individual SAMHD1 siRNAs (siRNA1, 2 and 4), a pool of 4 SAMHD1 siRNAs (siPool) or control siRNA (siControl). SAMHD1 was then detected on an immunoblot probed with rabbit anti-SAMHD1 antiserum. (C) BMDM were transfected with the pool of four siRNAs targeting mouse SAMHD1 (siPool) or control siRNA (siControl) and then infected with NL-Luc reporter virus 4 days later. After 4 more days, the cells were lysed and luciferase activity was measured. (D, E) BMDM were transfected with anti-SAMHD1 siRNA or control siRNA and, after 4 days, infected with HIV-1-CMV-GFP reporter virus. The cells were analyzed 4 days post-infection by flow cytometry. The result of 3 independent experiments is shown (D). Representative flow cytometry data from a single experiment is shown (E). (F) BMDM were transfected with SAMHD1 siRNA pool or control siRNA. After 6 days, extracts were prepared and the dATP concentration was quantified. These experiments were in triplicate with error bars to indicate the standard deviation of the mean.

Primary BMDM express more SAMHD1 than RAW264.7 and are nondividing terminally differentiated cells. Therefore, SAMHD1 might be expected to have a more pronounced effect on virus replication. To determine SAMHD1 antiviral activity in BMDM, we differentiated mouse bone marrow cells for 7 days in M-CSF and then transfected with individual or a pool of SAMHD1 siRNAs. After 4 days, we infected the cells with HIV-1 luciferase or GFP reporter viruses (Fig. 4A). To determine the efficiency of the knock-down, we lysed the cells the day of infection and quantified SAMHD1 on an immunoblot. The results showed that the individual and siRNA pool decreased the amount of SAMHD1 in the BMDMs (Fig. 4B). The luciferase reporter virus infected the SAMHD1 knock-down BMDM about 20-fold more efficiently than the control (Fig. 4C). The GFP reporter virus infected the cells knocked down with the SAMHD1 siRNA pool 8-fold more efficiently compared to the control cells. The individual SAMHD1 siRNAs also alleviated the block to infection although to a lesser extent (2.4-6.2-fold) (Fig. 4D, E). To determine whether the effect was mediated by an increase in the size of the dNTP pool, we quantified the dATP concentration in the cells. The results showed that SAMHD1 knock-down increased the dNTP pool 5-fold (Fig. 4F). These findings suggest that HIV-1 infection of primary mouse BMDM is restricted by SAMHD1 and that this is mediated by a decrease in the dNTP concentration.

### SAMHD1 is type-I and type-II IFN inducible in mouse BMDM and RAW264.7 cells

**Figure 5:**
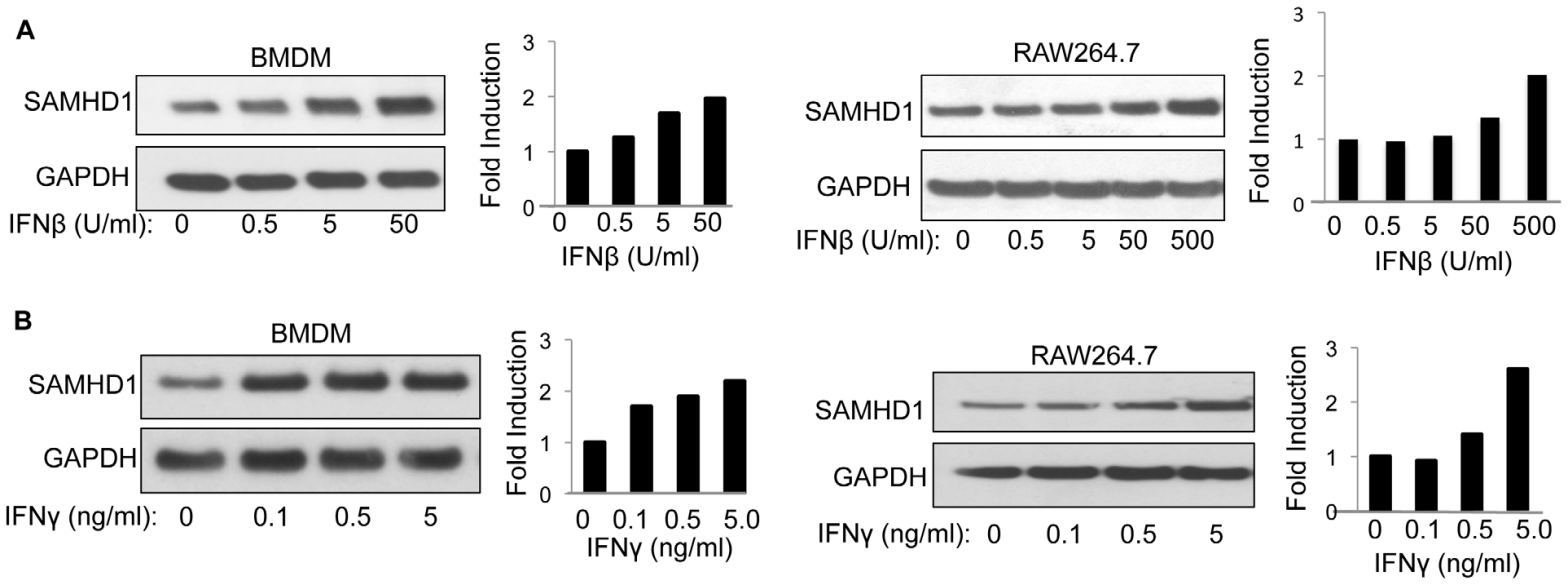
Mouse SAMHD1 is inducible by type-I and type-II IFN. (A) BMDM and RAW264.7 cells were treated for 24 hours with increasing concentrations of IFNβ. SAMHD1 was then detected on an immunoblot probed with anti-SAMHD1 antiserum, and GAPDH was detected as a loading control. The bands were quantified, and the quantification is graphed in the right panel. (B) BMDM and RAW264.7 cells were treated for 24 hours with increasing concentrations of IFNγ. SAMHD1 was detected and quantified as in (A). The data are representative of 3 independent experiments.

SAMHD1 was first identified in a screen for IFNγ inducible genes in mouse MDM [32]. In humans, it was shown to be induced by type-I IFN in specific cell-types. To determine whether mouse SAMHD1 is type-I and type-II IFN inducible, we treated BMDM and RAW264.7 with an increasing amount of mouse IFNβ or IFNγ and detected SAMHD1 on an immunoblot. We found that type-I and type-II IFN induced SAMHD1 in BMDM and RAW264.7 (Fig. 5A and B). The induction was 2-fold and was reproducible in three independent experiments. These results show that SAMHD1 is induced by type-I and type-II IFN in mouse BMDM.

### Mouse SAMHD1 regulates type-I IFN and ISGs

**Figure 6:**
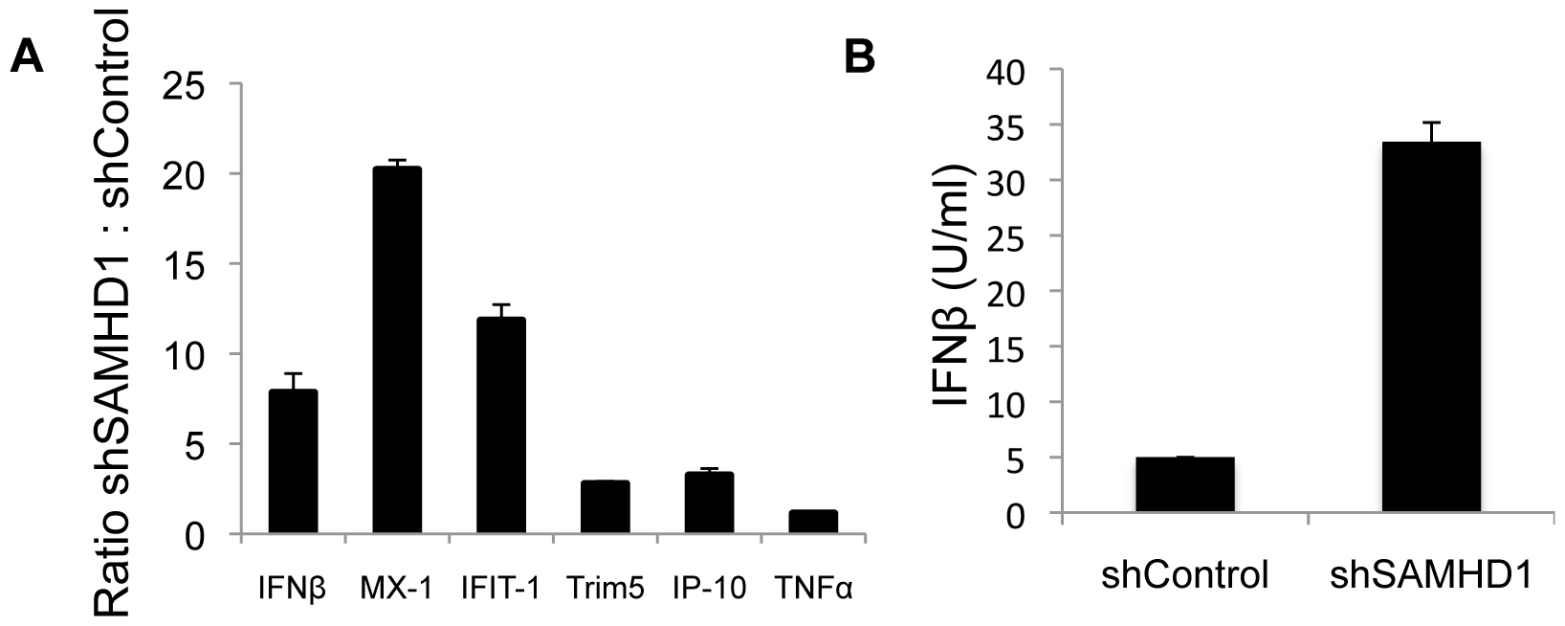
Type-I IFN is regulated by SAMHD1 in RAW264.7 cells. (A) Total RNA was extracted from SAMHD1 knock-down and control RAW264.7 cells. IFNβ, MX-1, IFIT-1, Trim5, IP-10 and TNFα transcript levels were quantified in triplicate by qRT-PCR, normalized to GAPDH, and the ratio (SAMHD1 knock-down cells to control cells) is shown. (B) Supernatant was collected from SAMHD1 knock-down and control RAW264.7 cells and IFNβ was quantified. Measurements were in triplicate and error bars indicate the standard deviation.

Polymorphisms in the gene encoding SAMHD1 in humans that cause AGS are associated with an increase in systemic levels of type-I IFN [26] suggesting that endogenous SAMHD1 suppresses the production of type-I IFN. To determine whether this is the case in the mouse, we quantified IFNβ mRNA transcripts in SAMHD1 knock-down and control RAW264.7 cells by q-RT PCR. The results showed that SAMHD1 knock-down cells had 7.8-fold more IFNβ mRNA transcripts than control cells (Fig. 6A) and 6.6-fold more IFNβ protein as determined by bioassay on L929-ISRE reporter cells (Fig. 6B). Induction of IFNβ is associated with an increase in the expression of ISGs. Quantification of the ISGs MX-1, IFIT-1 and Trim5 showed that these were also induced in the knock-down cells. IP-10 was also induced but TNFα remained unchanged.

## Discussion

The importance of SAMHD1 as a restriction factor for lentiviruses in primates has been clearly demonstrated, but its role in other mammalian species is not well understood. We present evidence that in the mouse SAMHD1 also plays a role as an antiviral defense. Knock-down of SAMHD1 in primary mouse BMDMs and RAW264.7 increased their susceptibility to infection with HIV-1 and MLV and increased the intracellular pool of dNTP. SAMHD1 was expressed in lymphoid tissues and in lymphoid and myeloid cells, similar to that of human. The enzyme was catalytically active in primary mouse tissues including BMDM, resting and activated T and B cells and RAW264.7 cells. In addition, SAMHD1 was induced by both type-I and type-II IFN, consistent with a role as a host restriction factor. SAMHD1 knock-down caused an increase in the production of type-I IFN and ISGs. The induction of IFN as a result of SAMHD1 knock-down was insufficient to induce an antiviral state, at least as detected using single-cycle reporter virus.

During the preparation of this manuscript, Behrendt *et al.* and Rehwinkel *et al*. reported results in which they generated a SAMHD1 null mouse and analyzed its susceptibility to retroviral infection [37], [38]. Behrendt *et al.* found that the null mice were more susceptible to HIV-1, and Rehwinkel *et al.* reported that the susceptibility of the mice to wild-type HIV-1 was unaffected but that they were more susceptible to infection by HIV-1 in which the reverse transcriptase was mutated to lower its affinity for dNTPs. Our results are consistent with those of Behrendt, further supporting a role for SAMHD1 in the mouse in restriction of wild-type retroviruses, both for HIV-1 and MLV.

In the mouse, SAMHD1 is expressed as two isoforms, a feature that differs from the human which expresses a single mRNA transcript. ISF1 conserves the carboxy-terminal CDK1 consensus target phosphorylation site (T634 in mouse) while in ISF2 this site is deleted by the splicing of the alternative 3’-exon. In the human, SAMHD1 is phosphorylated by CDK1 at T592 in cycling cells and this is thought to block its antiviral activity while not affecting its phosphohydrolase activity [18]. In the mouse, ISF1 could be similarly regulated but ISF2 cannot be phosphorylated and would be expected to maintain antiviral activity throughout the cell-cycle. Consistent with this mechanism, SAMHD1 maintained antiviral activity in cycling RAW264.7 cells against both HIV-1 and MLV. Thus, ISF2 in the mouse might provide a means by which cycling cells restrict virus replication, a capability that is absent in the human. We were not able to determine whether ISF1 is in fact phosphorylated due to the lack of a phosphorylation-specific antiserum.

Knock-down of SAMHD1 in RAW264.7 caused the cells to grow slightly faster, suggesting that reduction in the dNTP pool size by SAMHD1 limits the growth-rate of these cells. This finding differs from that in primary human fibroblasts where knock-down of SAMHD1 causes cell-cycle arrest as the result of the activation of a check-point that is sensitive to the dNTP pool size. RAW264.7 cells, a transformed cell line, appear to lack this check-point.

In AGS, defects in the genes encoding SAMHD1 or TREX1 cause the constitutive overproduction of type-I IFN. This situation is modeled in the SAMHD1 knock-down RAW264.7 cells. The knock-down cells constitutively produced an elevated level of type-I IFN and several ISGs, suggesting that SAMHD1 suppresses an intracellular signaling pathway that triggers type-I IFN production. SAMHD1 knock-out mice, although they do not have an AGS-like syndrome, have high levels of a large number of type-I ISGs [37]. The nature of the signal that is suppressed by SAMHD1 is not known. In the case of TREX1 deficiency, type-I IFN is induced by the accumulation of cytoplasmic DNA resulting from the failure to degrade endogenous retroelements [46]. In the case of SAMHD1 deficiency, the increased dNTP pool size could promote the synthesis of cytoplasmic DNA, triggering an intracellular nucleic acid sensor [47]. Alternatively, SAMHD1 is reported to have exonuclease activity and could degrade cytoplasmic DNA [20]. Arguing against this possibility, increased expression of endogenous retroelement RNA or glycoproteins was not detected in SAMHD1 knock-out mouse embryonic fibroblasts or BMDMs and the cells were not defective in their response to nucleic acid [38].

Recent findings in knock-out mice suggest a role for SAMHD1 as a host restriction factor to retroviral infection and our findings further support this conclusion. The absence of SAMHD1 in many cell-types suggests that it does not play a house-keeping role in nucleotide pool metabolism. The expression of SAMHD1 in lymphoid and myeloid cells may provide protection against infection of these cells as a means of ensuring that these critical cells are not targeted by viruses, protecting their role in the immune response. The identification of a cell-line that models the antiviral function of SAMHD1 and its role in regulation of the innate immune response provides a useful tool for further study of both pathways.
